# Regional transplant rates depend more on physician-dependent variables than on proximity to transplant center

**DOI:** 10.1007/s00423-023-02874-9

**Published:** 2023-05-12

**Authors:** Elnaz Payani, Nikolaus Börner, Dionysios Kolliogiannis, Stefan Brunner, Ingo Klein, Ursula Ehmer, Gerald Denk, Christian M. Lange, Klaudja Ograja, Peter Dietrich, Jens Werner, Markus Guba

**Affiliations:** 1 Department of General, Visceral and Transplant Surgery, LMU Klinikum Campus Grosshadern, Marchioninistrasse 15, 81377 Munich, Germany; 2 Department of Surgery, University Medical Center Regensburg, Regensburg, Germany; 3 Department of Surgery, University of Würzburg, Würzburg, Germany; 4 Internal Medicine II, Klinikum Rechts Der Isar, Technical University Munich, Munich, Germany; 5 LMU Klinikum, MED 2, Munich, Germany; 6 Transplant Center Munich, LMU Klinikum, Munich, Germany; 7 Transplant Center Erlangen-Nürnberg, University Hospital Erlangen, Friedrich-Alexander-University Erlangen-Nürnberg (FAU), Erlangen, Germany

**Keywords:** Disparities in liver transplantation, Proximity to liver transplant center, Liver transplantation, Socioeconomic factors

## Abstract

**Purpose:**

The objective of this work was to uncover inequalities in access to liver transplantation in Bavaria, Germany.

**Methods:**

For this purpose, the annual transplantation rate per 1 million inhabitants for the respective districts was determined from the aggregated postal codes of the place of residence of transplanted patients. The variables examined were proximity and travel time to the nearest transplant center, as well as the care category of the regional hospital. In addition, we assessed whether the head of gastroenterology at the regional hospital through which liver transplant candidates are referred was trained at a liver transplant center.

**Results:**

We could not demonstrate a direct relationship between proximity or travel time to the nearest transplant center and access to liver transplantation. Multivariate regression analysis shows that liver transplant training (*p* < 0.0001) of the chief physician (gastroenterologist) of the regional hospital was the most decisive independent factor for access to liver transplantation within a district.

**Conclusion:**

We show that the transplant training experience of the head of gastroenterology at a regional hospital is an independent factor for the regional transplantation rate. Therefore, it appears important to maintain some liver transplant expertise outside the transplant centers in order to properly identify and assign potential transplant candidates for transplantation.

## Introduction

Liver transplantation is the life-saving treatment option for patients with liver failure and hepatocellular carcinoma. However, the ever-increasing shortage of organs limits the widespread use of liver transplantation. It is therefore imperative to strive for equal access to liver transplantation and fair distribution of scarce donor organs.

Several reports from the United States (US) and United Kingdom (UK) describe unequal access to liver transplantation at multiple levels, in waiting list admission, delisting practices, and donor organ allocation [1–3]. In this context, race, gender, insurance status, but also proximity to the nearest transplant center were identified as factors for disparities [1–7]. In addition to transplant medicine, an association of geographic access to health care services and outcome was also observed in other health conditions, especially when prompt access to interventions is required [5, 8, 9]^.^ This one-sided important geographic proximity contrasts with the significantly better outcomes with centralized surgical procedures, e.g., pancreatic surgery and kidney and liver transplantation [10].

Since the health care and social security systems in the US and UK are not comparable with those in Germany, the results from these countries cannot be applied without restriction to Germany. In this paper, we therefore evaluated the influence of geographic proximity and simple hospital structure parameters on access to liver transplantation in Germany’s largest federal state, Bavaria.

## Material and methods

All patients transplanted in one of the 3 Bavarian liver transplant centers (LMU Klinikum, Munich; Universitätsklinikum Regensburg, Universitätsklinikum Würzburg) between July 2015 and November 2021 were included in the study. The postal codes of the patients’ place of residence were provided in anonymized form by the respective transplant centers. To preserve anonymity, the postal codes of patients were aggregated in the district of their residence. From these data, the annual transplantation rate per 1 million inhabitants was calculated for the respective districts. Population estimates and population density for each district were obtained from the census 2021 of the Bavarian State Office for Statistics. Urban was defined as more than 200 inhabitants/km^2^.

The longitude and latitude for the centroid of each of the 96 districts were entered into the Google Maps API. The shortest driving distance and travel time, unadjusted for traffic conditions, were calculated from the center of each district to each transplant center.

The level of care of each district hospital was extracted from the Bavarian Hospital Plan. The respective hospitals are categorized in the following service levels: (I) local basic services, (II) supraregional specialized services, (III) comprehensive and differentiated services (maximal care), and (IV) university hospitals (supramaximal care). Although we cannot exclude that a patient is treated in a regional hospital with an inadequate level of specialization, it is rather unlikely, since in Germany general practitioners refer patients to the nearest hospital with the best care for a particular medical condition. Patients with end-stage liver disease, who are potential liver transplant candidates, or patients who need advanced care, are referred to the head of gastroenterology at a transplant center.

In German health care system, the chief physician of a department has the medical directive, so whether patients are referred for further treatment depends largely on his assessment. Therefore, the chief gastroenterologists’ experience at a transplant center was considered indicative of transplant experience. This information was obtained from the publicly available CVs. The characteristic was fulfilled if at least 1 year of training was completed at a liver transplant center. In case of availability of several regional hospitals, the one with the highest level of care for this clinical profile was used.

### Statistical analysis

Normally distributed data are given as mean with standard deviation, and non-normally distributed data are given as median with interquartile range. Continuous variables were compared using the Mann–Whitney *U*-test and Student’s *t*-test, while categorical variables were analyzed using chi-squared test and Fisher’s exact test, as appropriate.

Independent factors associated with the regional transplant rate were identified using simple logistic regression analysis and receiver-operating curves. In a multivariate analysis, we used multiple linear regression. The dependent variable (*y*) used was the annual transplant rate per 1 million inhabitants per district. *R*2 describes the fraction of all variance in *y* that is explained by the multiple regression model and always ranges between 0 and 1. A result was considered significant when *p* < 0.005.

All statistical analyses were performed using SPSS version 25 and GraphPad Prism version 9.0.0. Mapping was performed using paintmaps (https://paintmaps.com).

The study adheres to the RECORD guidelines, as reflected on the EQUATOR website Mapping.

## Results

In Bavaria as a whole, the annual liver transplant rate per 1 million inhabitants was 7.7 ± 0.5. There were no significant differences in the annual liver transplantation rate between the 7 governmental districts in Bavaria (*p* = 0.64) (see Table 1).

**Table 1:** Basic characteristics of the study population

Parameter		
Total LTx/center	LMU-Klinikum Munich	357
	UK Regensburg	218
	UK Wurzburg	77
LTx/1 million inhabitants/year (Governmental districts, mean ± SEM)	Upper Bavaria	8.6 ± 0.8
	Lower Bavaria	7.6 ± 1.7
	Upper Palatinate	9.4 ± 1.4
	Upper Franconia	6.6 ± 1.5
	Middle Franconia	7.0 ± 0.9
	Lower Franconia	8.1 ± 1.4
	Bavarian Swabia	6.4 ± 1.3
Proximity to the next transplant center (km; mean interquartile range)		79 (60.3;106.8)
Travel time to the next transplant center (min; mean interquartile range)		60 (50;80)

**Fig. 1:**
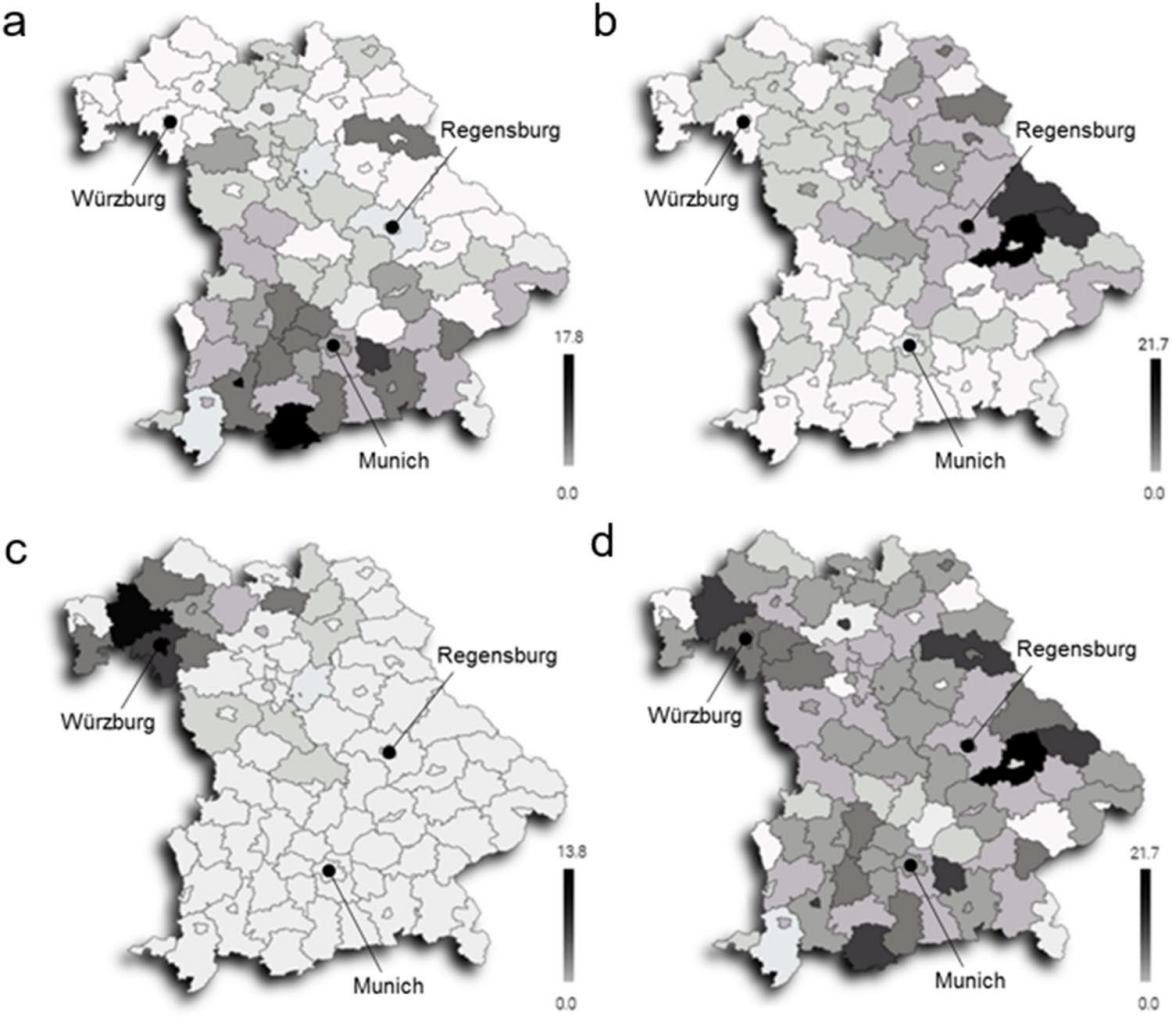
Choropleth map liver transplants/1 million inhabitants/year within a district. Per center: **a** Munich (established care network with university hospitals Erlangen and Rechts der Isar of the TU Munich),** b** Regensburg, **c** Wurzburg, **d** all centers

The transplant rates on district level are visualized in Fig. 1. The analysis of patient flows for the respective centers shows that not all patients necessarily choose to go to the nearest transplant center but may travel further afield. A formal care network with the university hospital in Erlangen may explain the outreach of the center in Munich. A lower transplantation rate seems to be present in the border area of the center service areas. Apart from that, no particular distribution patterns and no significance with respect to the transplantation rate were apparent (*p* = 0.21).

The median travel distance to the nearest transplant center was 79 km, with 75% of all patients within 100 km of a center. The median travel time by car for a patient was 60 min, 75% of all patients were able to reach a transplant center within 80 min, and the longest travel time was 120 min.

**Fig. 2:**
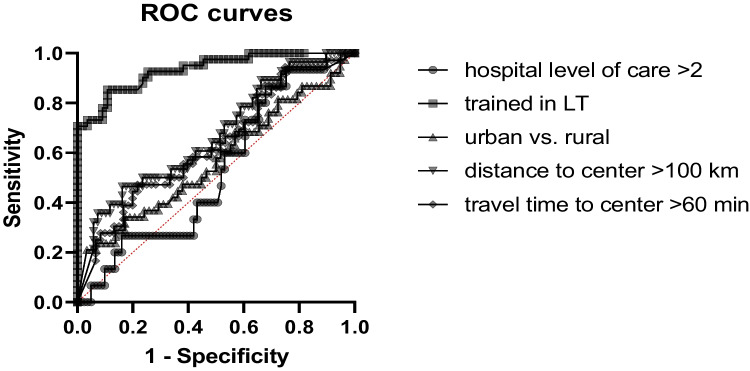
ROC curves for categorical variables based on the annual transplant rate per 1 million inhabitants per district

Simple logistic regression analysis for categorized variables showed no significance for rural versus urban areas (*p* = 0.14), hospital service category of the regional health provider (*p* = 0.69), or proximity and travel time to the nearest transplant center (*p* = 0.41). In contrast, past liver transplant training of the responsible chief physician of the regional hospital showed a high correlation with the annual transplant rate of the district (*p* < 0.0001). ROC curves for these variables based on the annual transplant rate per 1 million inhabitants per district are shown in Fig. 2.

**Fig. 3:**
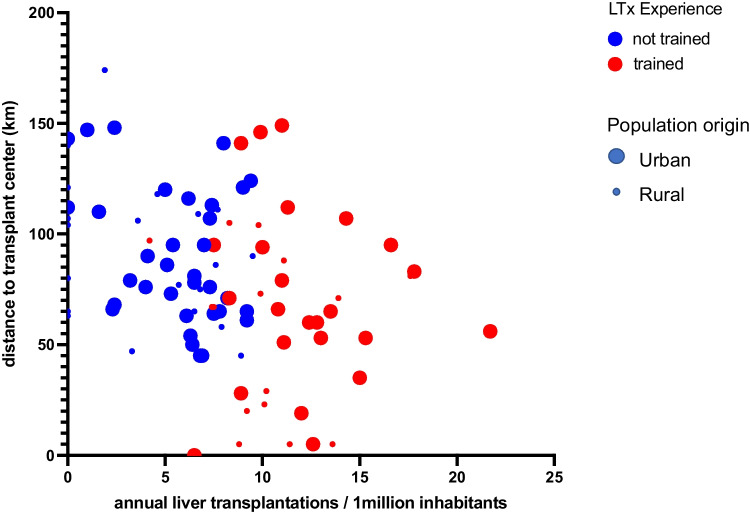
Bubble plot visualizing the multiple linear regression analysis shows the strong correlation between liver transplant training of the referring physician and the annual liver transplant rate. Blue bubbles represent “no LTX training” red bubbles “LTX trained”; small bubbles “rural” and large bubbles “urban” residence

Multivariate regression analysis show that liver transplant training (*p* < 0.0001) was the most decisive independent factor for access to liver transplantation within a district (see Table 2). Hospital levels of care have been categorized into maximum care facility (levels 3 and 4) and regular care facility (levels 1 and 2). Fig. 3 visualizes the significance of liver transplant training of the referring physician. Overall the regression model presented a goodness of fit with an *R*-squared of 0.5962.

**Table 2:** Multivariate regression analysis for annual liver transplant rate per 1 million inhabitants per district

Variable	Parameter estimates (beta)	Standard error	95% CI (asymptotic)	*P* value
Maximum care facility	− 0.3999	1.006	− 2.398 to 1.599	0.69
LTX experience	6.215	0.7365	4.752 to 7.679	< 0.0001
Urban residency	1.768	0.7120	0.353 to 3.183	0.14
Distance to TX-center (km)	− 0.01351	0.01641	− 0.046 to 0.019	0.41
Distance to TX-center (min)	− 0.006683	0.02404	− 0.054 to 0.041	0.78

*CI* = confidence interval, *LTX* = liver transplant, *TX-center* = transplantation center

## Discussion

Liver transplant services in Germany were not developed prospectively to take into account the epidemiology of liver disease or differences in disease burden or complexity. Nor were they developed to efficiently map patient needs in a logical geographic distribution. Instead, liver transplant services historically developed primarily at university hospitals, reflecting the ambition of academic surgeons and institutions rather than a business assessment of need.

In Germany, 21 active liver transplant centers currently serve a population of 83 million. In the state of Bavaria, there are 3 active centers that serve approximately 13 million people [11]. The situation in Bavaria is therefore a good approximation of the situation in Germany as a whole to address the question of which geographic and variables influence access to liver transplantation.

The key finding of our analysis is that access to transplantation is largely determined by the referring head physician at the regional hospital rather than by the level of care provided by the hospital or the distance to the nearest transplant center. The only independent factor for referral and thus access to liver transplantation was whether the chief physician of gastroenterology of the local hospital had completed at least parts of his training at a liver transplant center.

For further understanding, it is necessary to look at the peculiarities of the German health care system. First of all, there is no dedicated specialty for hepatology, so the care of liver patients is usually provided by physicians with a background focused more on gastroenterology. Assignment to a transplant center is not formalized but is essentially left to the referring physician. Chief physicians have the directive of action for their department for medical issues. For this reason, it seemed viable to us to base the analysis on the training history of the chief physician (gastroenterologist). From our point of view, it seems reasonable to assume that referring physicians who do not have hands on experience with liver transplantation might be more reluctant to refer a patient for liver transplantation. In addition, misconceptions might also exist regarding regulatory conditions, exclusion criteria, and the likelihood of success of this very complex treatment modality. In this context, Loy et al. show for referral patterns for alcohol-related liver disease that physicians without liver transplant center training were more likely to require longer duration of sobriety prior referral [12]. The influence of training and experience in a subspecialty on the assignment of appropriate therapy is also observed in other fields of medicine [13].

Indirectly, the filling of chief physician positions is also related to the distance to the nearest transplant center. Thus, chief physician positions in or near attractive metropolitan areas are more popular among applicants with a university background than remotely located smaller hospitals. Neither the distance to the nearest transplant center nor the travel time had an effect on access to liver transplantation. Yet, it is quite understandable that the distance to the nearest transplant center may play a role in countries with a larger area coverage or fewer transplant centers. Several studies from the US and UK show this relationship for access to transplantation, but also for outcomes after transplantation [1, 2]. Overall, it should be noted that with the distance to the nearest transplant center, which is usually located in a larger city, many socioeconomic variables and variables inherent in the respective health care system may also change, without the respective relationship being immediately apparent. In this context, however, our analysis shows no relationship between access to transplantation and the population density of the patient’s place of residence or the care category of his or her regional hospital.

The broader implications of our study are that regional care structures need to be strengthened to ensure access to liver transplantation everywhere, regardless of prior liver transplantation experience of the referring physician. In the USA, good experiences have recently been made with a virtual transplant center to ensure the access of remote patients [14]. The integration of regional health care services through appropriate network and satellite arrangements has been described in great detail by O`Grady [15]. In fact, we started such a satellite arrangement with the University Hospital in Erlangen 6 years ago, which allows for high-quality care for liver transplant patients close to home before and after transplantation. Special education sessions in first-care hospitals could raise awareness of the benefits of liver transplantation.

Our data do not suggest that the current approach of centralizing liver transplantation should be abandoned, because sheer distance to the nearest transplant center was not directly related to access to liver transplantation and the benefits of centralization outweighed those of more convenient reachability [10].

As with any observational study, unidentified confounders may limit this significance of the study. As presented in the multivariate analysis due to the involved human behavior a precise prediction model appears difficult to achieve. Furthermore, with limited data sets available, we cannot exclude that differences in the socioeconomic status or disease incidences, although unlikely, have an impact on the results. In our experience, the flow of patients will mainly reside within Bavaria, with only 2 districts in the south and northwest of Bavaria expected to see significant referrals to neighboring states. Even if the liver transplant training of the chief physician cannot be considered an absolute criterion for the experience of the servicing hospital, it is at least an ascertainable characteristic and, in our experience, a good surrogate. Furthermore, our study is not free from sampling bias. We do not present the patients referred or listed for a liver transplantation, which would indeed present even more insight. Since these data are not completely available, we provided the number of transplanted patients, from whom we have detailed and well-documented data.

## Conclusion

We show that the transplant training experience of the head of gastroenterology at a regional hospital is an independent factor for the regional transplant rate. Therefore, it appears important to maintain some liver transplant expertise outside the transplant centers in order to properly identify and assign potential transplant candidates for transplantation.

## Data Availability

The data that support the findings of this study are available on request from the corresponding author. The data are not publicly available due to privacy and ethical restrictions.
